# Planking or the “Lying-Down Game:” Two Case Reports

**DOI:** 10.2196/ijmr.6568

**Published:** 2017-03-23

**Authors:** Stefania Barbieri, Paolo Feltracco, Luca Omizzolo, Rossella Snenghi, Rafi El Mazloum, Gianna Vettore, Mauro Bergamini, Armando Stefanati, Daniele Donato, Cecilia Ferronato, Francesco Maria Avato, Alberto Tredese, Rosa Maria Gaudio

**Affiliations:** ^1^ Preventive Medicine and Risk Assessment University of Ferrara Ferrara Italy; ^2^ Department of Urgent and Emergency Care University of Padova Padova Italy; ^3^ Forensic Medicine and Toxicology University of Ferrara Ferrara Italy; ^4^ Department of Legal Medicine University of Padova Padova Italy; ^5^ Department of Directional Hospital Management Padova University of Padova Padova Italy

**Keywords:** planking, the lying down game, serious games, blunt trauma, multiple trauma, social networks, health care costs

## Abstract

**Background:**

The monitoring and management of risks regarding children and young people admitted to the emergency department as a result of dangerous behaviors distributed via the Internet should be based on clinical reasoning and knowledge about these social media–related phenomena. Here we examine 2 cases of teenagers who reported severe injuries while performing the “planking” craze, a challenge that consists in lying face-down stiffly like a board on any kind of surface.

**Objective:**

Our objective is to examine and describe the Internet craze called planking, also known as the “lying-down game,“ through 2 case reports from our experience, enriching this study with information gained through discussions with secondary school teenagers.

**Methods:**

Details of the 2 case reports were taken from electronic medical records giving information on care support processes, care management, and the costs of traumatic episodes. Demographic data, hemoglobin and serum lactate values, and Injury Severity Scores were evaluated. The study took place in secondary schools of our city from 2013 to 2014 during medical education courses, with the aim of analyzing the influence of social media on teenagers' activities and behaviors.

**Results:**

Both patients suffered multiple trauma injuries and needed high-level health assistance. The first patient underwent a splenectomy and the second one a nephrectomy; both of them required a long hospital stay (14 and 20 days, respectively), and the costs for their management have been estimated at US $27,000 and US $37,000, respectively. Their decision to perform the planking in dangerous locations was due to their ambition to gain peers' acclaim through shared videos and pictures.

**Conclusions:**

Reporting and understanding these cases may potentially help prevent future events occurring in similar circumstances: the scientific community cannot leave this problem unaddressed. There is also a role of education resources for health care professionals; for this, we must identify and follow up strange or misleading information found on websites. A key element of this research study was to report physicians’ misperceptions concerning planking and, with these cases used for teaching purposes, improve knowledge of the clinical and forensic aspects of this emerging problem.

## Introduction

**Figure 1:**
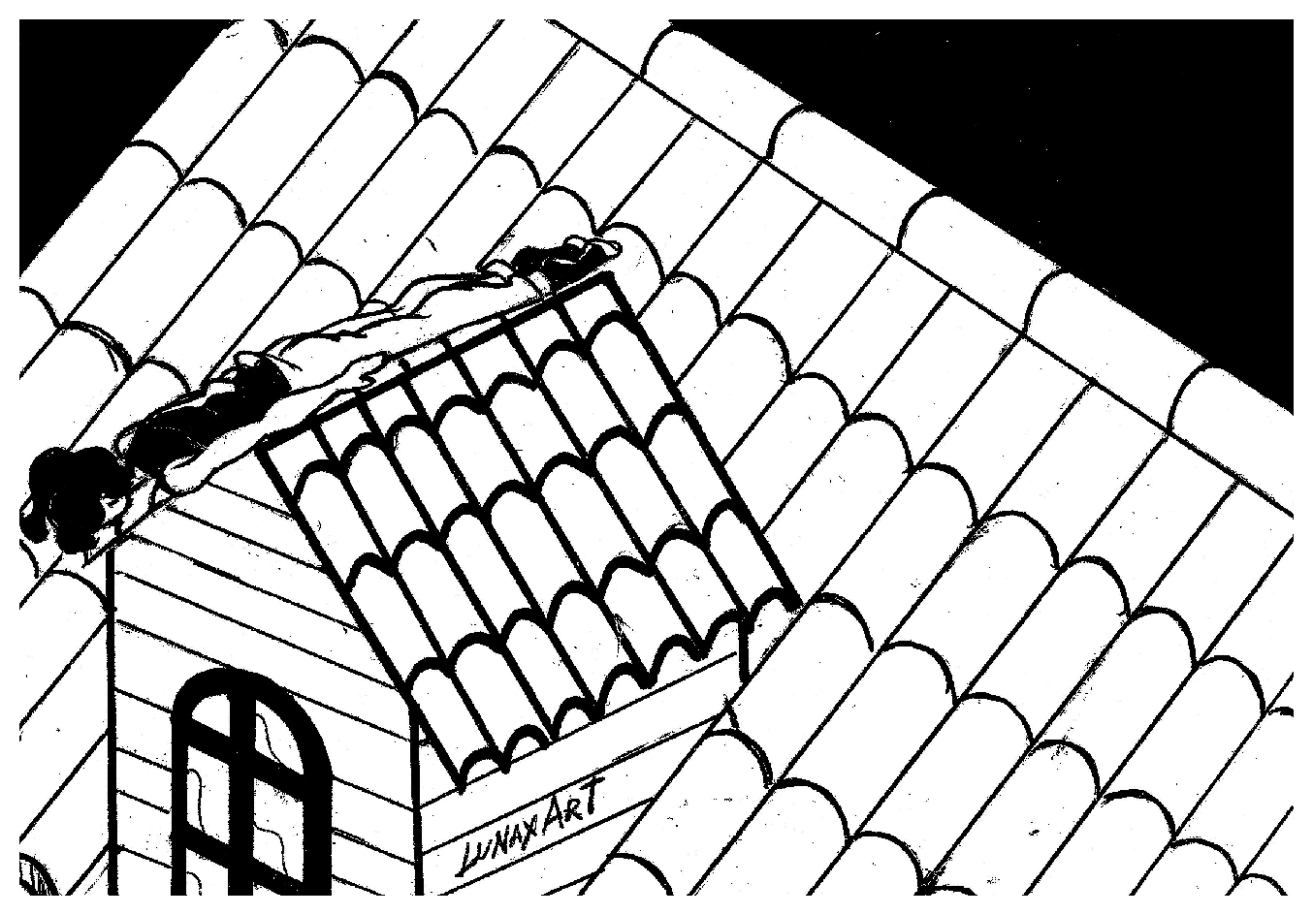
Planking on a rooftop.

**Figure 2:**
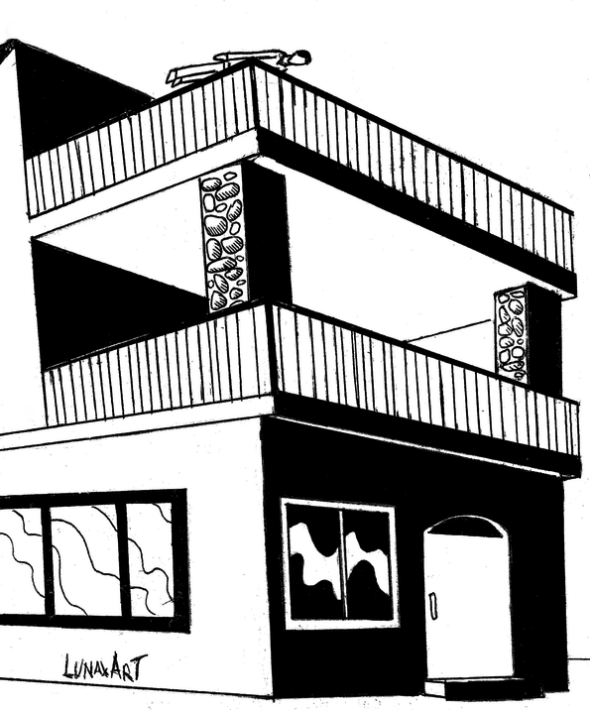
Planking on a balcony railing.

The emergency department (ED) these days must also examine behavioral changes: our real challenge is to understand them within the sphere of programs for surveillance, research, and innovation. Preteen children and adolescents are the most frequent users of social networks, blogs, and forums of all kinds. The monitoring and management of risks in treating children and young people admitted to the ED as a result of dangerous behaviors spread by the Internet should be our basis for clinical reasoning. Medical decision making must be developed to deal with a specific problem: knowledge of a new practice by young people which may result in serious injuries due to multiple independent risk factors following falls from various heights and in different positions, etc (position, place, biomechanical characteristics, mechanism of injury). The 2 cases described here are not attempted suicides; descriptions of the accident scene show nonfatal falls from heights by 2 young men (Figures 1 and 2).

A key element of this research is to report physicians’ misperceptions concerning planking and, using ED data, improve knowledge of the clinical and forensic aspects of this emerging problem. The context was developed by the authors according to clinical, forensic, and health care experiences including previous experiences of training at various graduate and postgraduate levels and with appropriate multidisciplinary input from experts in medical education, focusing on defining isolated but new social activities aimed at increasing young people’s image of themselves on social network sites by gaining likes from friends and visitors to their profiles [1-4]. A “like” is an action which can be made by a social media user (Facebook, Instagram, etc): instead of sending a message or a status update, the user can click the like button as a quick way of showing approval and sharing the message. Scores are calculated with a great number of variables, including the number of followers and friends of each person, the frequency of updates, and the number of likes, retweets, and shares that each person receives [2,4]. High scores are linked to the level of influence and are calculated according to positive or negative feedback from the target audience, especially as regards increasing the number of likes which represent each user's profile [5-8].

Most adolescents use online networks to increase their knowledge regarding games, videos, culture, scientific knowledge, fact-related reproductive health, wellness programs, etc, but negative effects on mental health such as cyberbullying, sexting, and increasing the number of friends through blogs, photos, videos, sharing, or real-life background connections have been reported [8-10]. Social networks may be considered as new ways of communication which can influence individuals’ lifestyles, either positively by gaining likes or negatively by losing them. The photos of young people during planking are unusual in both pose and situation and sometimes have the greatest effect when they are posted on the Web by young people simply because they believe they will attract new likes to their page through creative, funny, or crazy photos or videos [10-11].

At present, there are no official reports in the literature of lesions due to trauma as a result of planking, which has probably had little effect on immediate trauma fatalities, but these results can be projected to other trauma centers and processed to create injury surveillance data. Facebook is one of the most popular social network services with more than 1 billion daily active users around the world [1]. The goal of the adolescents is to upload videos, photos, and personal details with the intention of creating a self-descriptive profile. Social networking sites offer new social contact and knowledge of other people’s attitudes and behavior mediated by, for example, the Facebook platform, but further exploration and developed strategies are necessary to understand when the interactive functions include high-risk behaviors and when they represent an opportunity to establish modern challenges through blogs, wikis, or posted contents. A recent review confirms Facebook’s potential for the study of human behavior [3].

**Figure 3:**
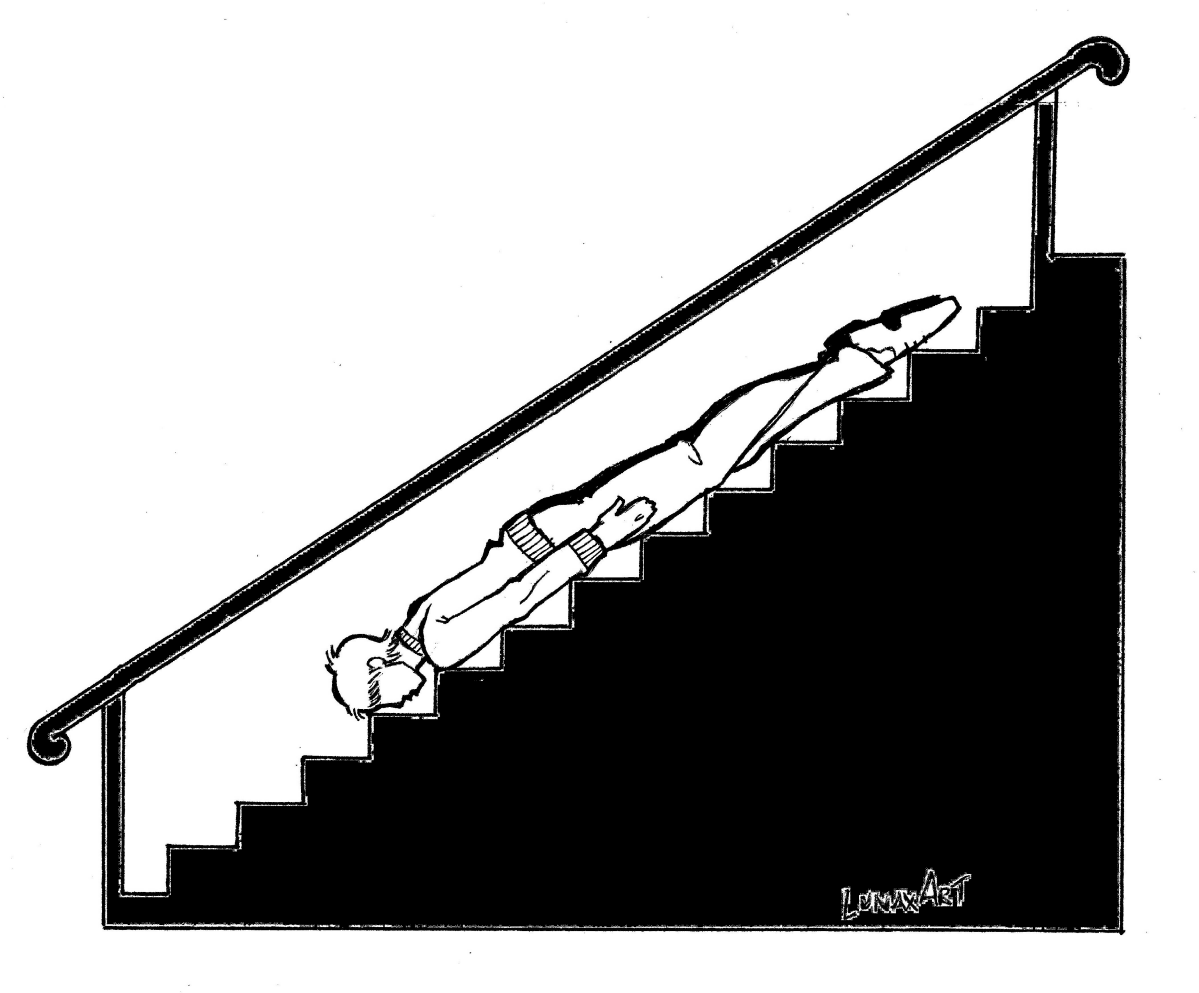
Planking or the lying-down game.

Planking consists of lying face-down, stiffly like a board, on any kind of surface (Figure 3). Participants have photos taken of themselves and upload them via the Internet in order to obtain a high number of likes on their profiles. Most cases of planking do not involve injury because the practice is rarely dangerous and usually performed in safe areas. However, adolescents often choose unusual and sometimes dangerous places in order to draw more attention and increase their number of likes.

The following case reports describe the patterns of injury and their severity in 2 cases of planking which resulted in traumatic lesions due to vertical deceleration. The literature contains some data of clinical series of children and adolescents admitted to EDs after falls from a height (>5 meters) or due to height trauma for various reasons (attempted suicide, dyads, homicide, accidents), the severity of injuries, and outcomes. However, surprisingly, we could not find any report on the pathology of trauma resulting from falls from a height in relation to planking [12-15].

## Methods

Details of the 2 case reports were taken from electronic medical records giving information on care support processes, care management, promotion of public and population health, and the costs of traumatic episodes. Demographic data, hemoglobin and serum lactate values, and Injury Severity Scores (ISSs) were evaluated. ISSs, an essential index of injury severity related to the risk of mortality, are reported to emphasize trauma pathways and costs. New opinions are introduced by physicians for additional care processing so that this preliminary health information could improve our knowledge of health care. The study took place in secondary schools of our city from 2013 to 2014 during medical education courses; during the lessons we analyzed the integration of social media on adolescents’ activities and behaviors through discussions held in small groups of students with the authors’ supervision. The results indicate that both multimodality and interactivity contribute to educational outcomes individually. Implications for educational strategies and future research directions have been discussed in previous studies [6-10].

## Results

Internet profiles and information supplied by friends helped to determine the reasons for the place and position of the patient’s fall, details of behavioral data, and any clinical effect of planking. Both accidents were the result of planking scenarios enacted by young people to enhance their status with their peers and included sharing pictures or videos through social networks. This attitude can be imitated by those who are deeply influenced by network sociality [5] and who feel challenged to undertake ever more extreme acts. The increasing popularity of photos in planking positions reveals the causes of injuries. We describe the cases of 17- and 18-year-old males who arrived at the ED with blunt abdominal and thoracic trauma injuries after planking accidents. Both patients were stable on arrival at the ED.

### Case Report 1

**Figure 4:**
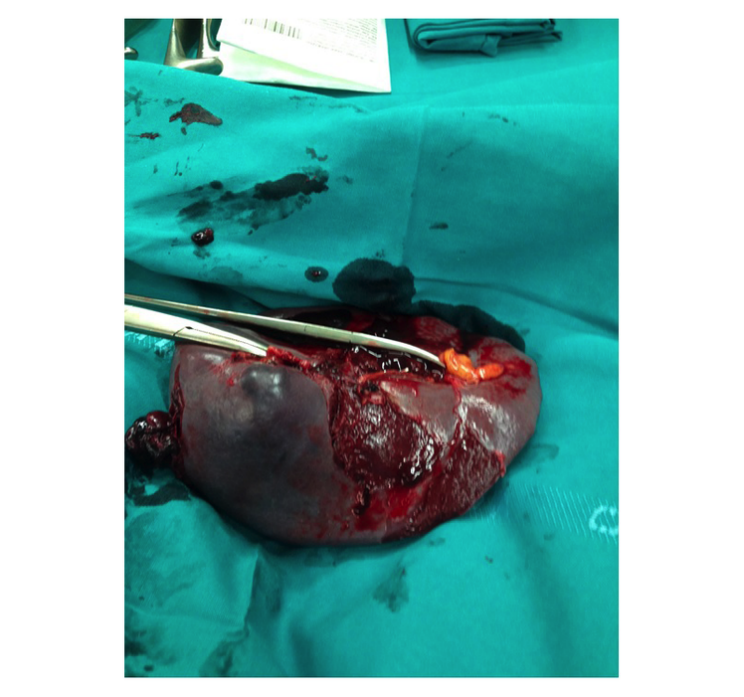
Spleen removed through laparotomy.

An 18-year-old Italian boy was admitted to hospital after an accidental fall from a height of 5 meters. Neurological assessment revealed a Glasgow Coma Scale score of 14/15. On admission, blood pressure was 74/45 mm Hg, pulse 145 beats per minute, respiratory rate 32 breaths per minute, and hemoglobin concentration 8 g/dL. The report refers to an accident in which the boy was planking over a balcony; he suddenly lost his balance and fell from a height of over 5 meters, first onto a canopy, which broke his fall to a certain extent, and then a further 2 meters to the ground. The dynamics were precipitation and the boy’s semilateral or lateral left decubitus position during the impact. According to the splenic injury scoring system of the American Association for the Surgery of Trauma, the patient suffered a type III injury, with a subcapsular hematoma exceeding 50%, intraparenchymal hematoma exceeding 2 cm, and a 3-cm laceration through the splenic parenchyma. Classification of splenic injury was based on the rigorous definition of anatomic disruption [16]. Radiographs from the initial examination, which included chest, pelvis, and lateral and oblique cervical spine, were assessed together with radiographs of the specific sites of injury followed by laparotomy for blunt injuries. Abdominal sonography for trauma was used to investigate the splenic injury in the abdomen due to freed blood, and computed tomography (CT) scans were then taken. One hour later, due to sudden hemodynamic instability, sonography was repeated and found positive for trauma; surgical exploration was then decided upon. The subcapsular hematomas and parenchymal disruption of the spleen (Figure 4) were not discovered by ultrasound and did not result in a significant hemoperitoneum, but the subsequent focused abdominal sonography for trauma (Eco-FAST) scan with intravenous contrast helped diagnosis. Because of ongoing hemodynamic lability, the patient underwent emergency laparotomy. The severity of the case included blood accumulating in Morrison’s pouch and in the pelvis and injury to the pancreas. The length of hospital stay was 14 days. The costs for patient 1, although trauma is generally underreported and depends on its severity, were €25,600 (approximately US $27,380) including laboratory and radiological work, intensive care unit stay, operating theater surgery, dialysis, and total costs of hospitalization.

### Case Report 2

A young male aged 17 was admitted to the ED. Initial vital signs were blood pressure 120/59 mm Hg, heart rate 133 beats per minute, respiratory rate 16 breaths per minute, and oxygen saturation as measured by pulse oximetry 97% on a nonrebreather mask. In the ED, vital signs were blood pressure 75/45 mm Hg, heart rate 145 beats per minute, respiratory rate 22 breaths per minute, and oxygen saturation (as above) 95%. Abdominal ultrasound and contrast-enhanced dynamic CT revealed a large retroperitoneal hematoma. The patient was submitted to surgery immediately and a left nephrectomy for acute hemorrhage due to full thickness perihylar laceration was performed. The mechanism of damage consisted of blunt renal trauma resulting from sudden deceleration, which affected the renal parenchyma and the vascular pedicle. This deceleration and the resulting hyperextension resulted in laceration and partial avulsion of the kidney at its proximal point of fixation. A preexisting renal abnormality decreased the possibility of salvage.

**Figure 5:**
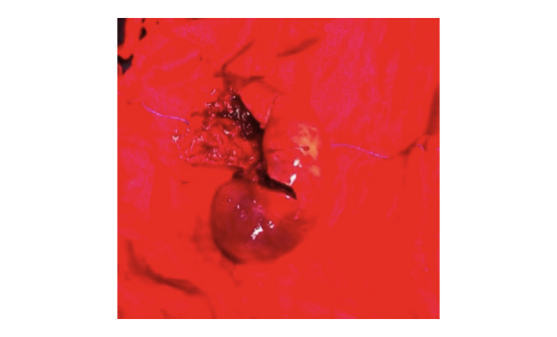
Full thickness renal perihilar laceration.

Figure 5 shows the parenchymal laceration extending through the renal cortex [17]. Hospital stay lasted 20 days. For patient 2, costs amounted to €35,000 (approximately US $37,440) including laboratory and radiological work, intensive care unit stay, operating theater surgery, dialysis, and total cost of hospitalization.

## Discussion

### Principal Findings

Trauma due to a fall from a height is a particular type of blunt trauma produced by rapid vertical deceleration and impact forces [12]. Such cases may include cervical spine fractures associated with other fractures of the thorax, scapula, bilateral upper arm, and/or pelvis [13-14]. Further internal damage may lead to delayed splenic rupture. Contusion of the spleen is characterized by the capsule filling with blood, and the opposite is true in the case of the kidney. Cases of spleen and kidney contusion differ greatly, due to their differing capsules; that of the spleen is thicker than that of the kidney, so blood keeps filling the capsule, producing a subcapsular hematoma. The cases reported here were critical but nonfatal accidents following falls by adolescents, representing “crazy” adolescent and young people’s behavior which may culminate in severe injury. See Textbox 1 for cases in which the exact location of planking may be a dangerous predisposing factor in determining unintentional injury.

**Location of planking accidents.**

- Case report 1: Patient was planking on a roof, lost his balance, bounced off a canopy, and fell a total of 10 meters.
- Case report 2: Patient lost his balance, fell from a second floor balcony, turned over in the air and landed on his back, falling a total of 7 meters.

**Figure 6:**
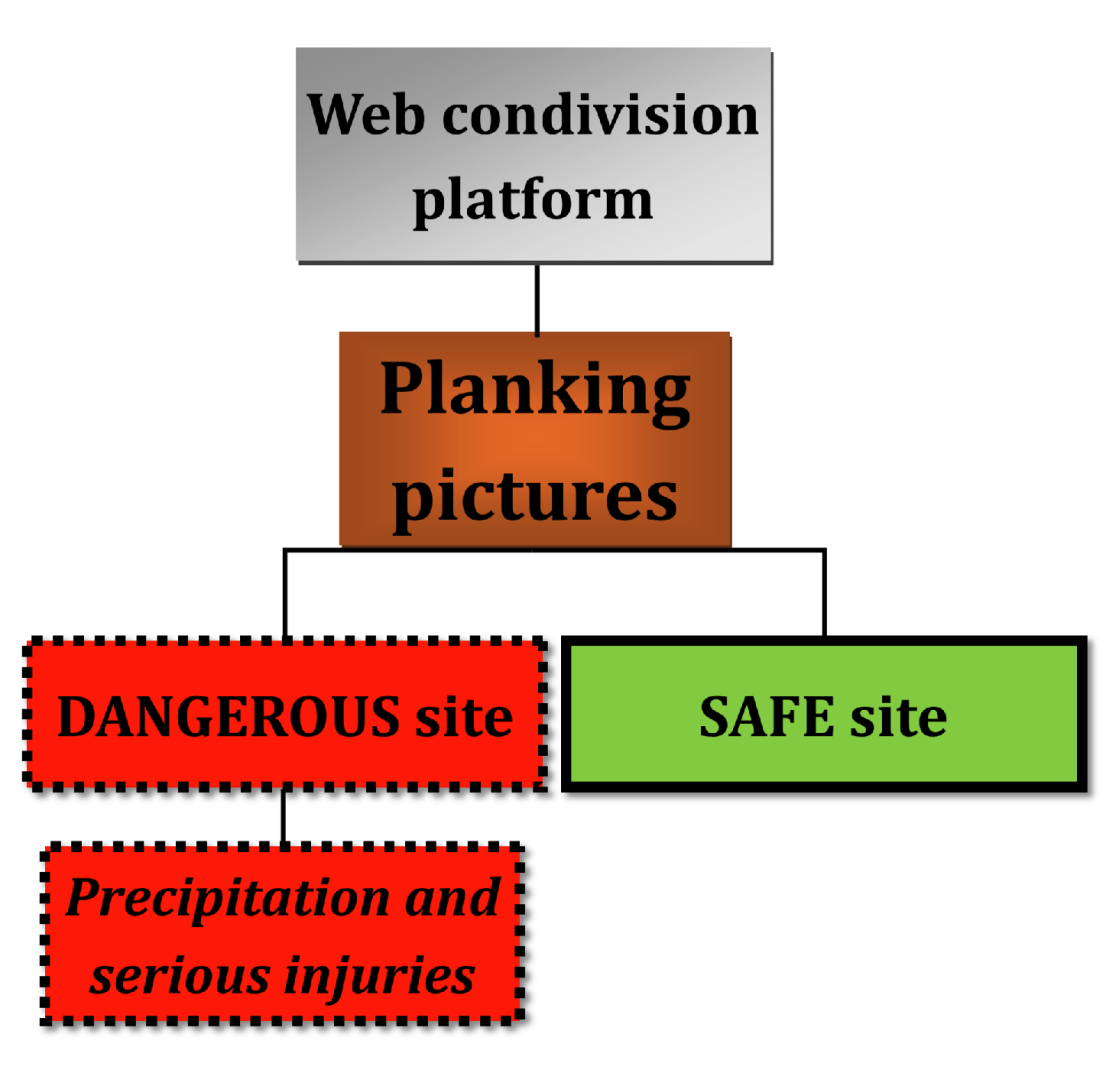
Planking may be performed in both safe and unsafe locations, the latter being associated with falls and injuries.

**Figure 7:**
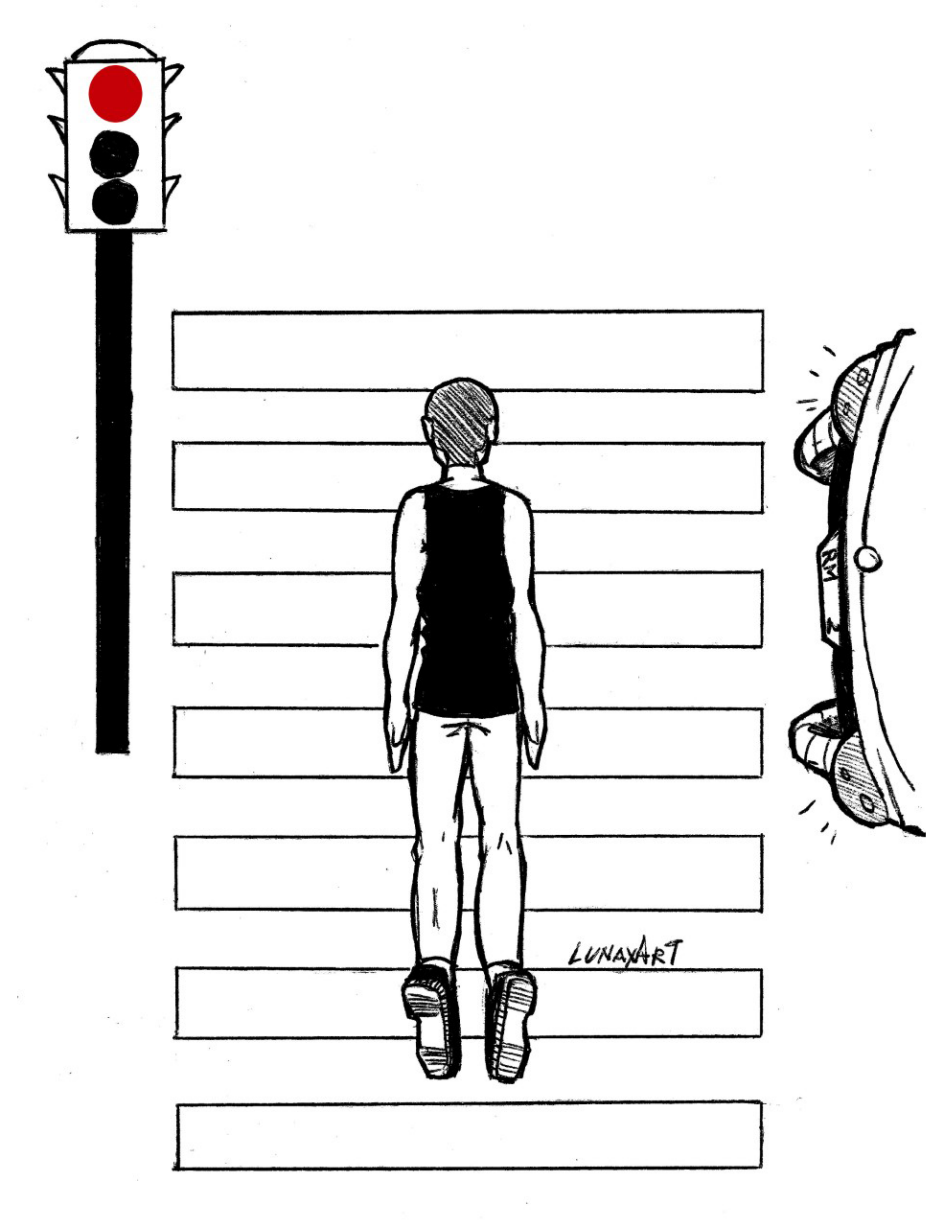
Planking on the street.

The importance of this point allows us to reflect more widely on various aspects of adolescents’ daily lives and lifestyles (Figure 6). These cases are focused on a specific context of wider academic research, and they suggest and support the development of a new important dimension for unanswered questions on the pitfalls of social network during dangerous games. Ongoing audit will assess the impact and safety of the new blunt trauma related to planking phenomenon, new Web-based alcoholic games, biker roulette games, and the other challenge activities spread through the Web by the adolescents’ virtual communities [6-10]. These samples are somewhat representative of most of the target population, and they represent an opportunity for future improvements in scientific works and performances of physicians, nurses, and sociologists. In writing this paper, we aimed at better understanding of the consequences of planking, which may be dangerous if it is done at heights or in potentially dangerous places (eg, higher than 5 meters or in or on such places as train tracks, crosswalks, public transport vehicles, canopies, terraces, balconies, roofs, curbs, street furniture). Adolescents feel the need to communicate emotions and actions, sometimes by performing rituals based on dangerous actions, in order to strengthen their social bonds with their peers. Our cases were similar to other reports describing patients after precipitation and were diagnosed as severe trauma. Based on a MEDLINE search of literature in English from 2000 to 2014, to the best of our knowledge, ours is the first case of trauma related to planking ever reported. Planking can be done in various ways, either by lying face down safely, or dangerously, perhaps while lying somewhere high up (descriptions of planking sites can provide important information on how the trauma-related injuries occurred, as in our 2 cases). The consequences of damage to the described organs generally reflect the magnitude of the height of fall, associated with extensive fractures of the upper and lower limbs and even more severe visceral injury to internal organs by direct impact [13]. Some activities are used as strategies to increase the number of followers, in line with the popular expression “big likes are on your mind day and night.” For the new generation of adolescents, being popular means not only doing something that makes you appear older, stronger, and cooler than your friends in real life but also in virtual life on social networks, which are often equally important to adolescents in our society today. Good documentation of medical records is essential for reasons of economics. This study aims at enhancing greater insights in emergency and medicolegal teams, together with more knowledge about the influence of social networks on health care, which will expand to become an integrated clinical practice [6-10]. Planking is a relatively new phenomenon and has already attracted the interest of many adolescents [8]. Proliferation of new activities and games, presented in videos via the Web, can influence adolescent behavior; in planking, they result in photos in which an individual lies face down in unusual public spaces (Figure 7).

The Klout score is tangible proof of the effect of the Internet on adolescent lifestyle; this social network service offers tailored statistical analysis of social media. In particular, it estimates the influence of users through a score (0-100), ranging from the degree of interaction in user profiles of similar popular sites (eg, Twitter, Facebook, Google+, LinkedIn, Foursquare). A Klout score can be obtained on the extent of the network, its users, the content generated, and the feedback level obtained [9]. The cases we describe essentially define how planking may be dangerous; we report the possible severity of planking-related injuries and identify specific accidents and influencing factors. It is difficult to see how effective prevention measures could be defined, although restricted access to certain websites may be one option. Our findings have important implications in terms of insurance and changes in cause-specific injuries and intent-specific groups which may reflect differences in trauma coding. Intentional and unintentional injuries due to planking are more likely to be seen by ED personnel, although there are differences in how trauma data is coded (misclassification of cause-specific and intent-specific injuries). Improving the documentation of the circumstances of an injury-causing event is essential for prevention purposes, and many new categories could be added for falls: these 2 facts have particular implications for injury prevention. The safety of ED care has been identified as strategic in clinical practice in children and adolescents. There are few epidemiological reports in the literature, compared with the amount of data available on adults falling from heights, and proper comparisons of experiences and solutions applied in varying organizational contexts is urgently needed [12-14]. In this work, the direct costs of the 2 accidents are described: the costs of treatments in the trauma room, any fluid and blood replacement therapy, surgeries, treatments in the intensive care unit, and the human capital approach. This value is calculated based on individual injuries, but a standardized approach to economic evaluation is needed to further prioritize mainly regarding the investing in injury prevention. This study does not compare the costs for these cases and the economic aspects of trauma-related planking, but the authors propose to examine this in detail in future studies.

### Limitations

This study has several potential limitations. First, these 2 participants were interviewed once but we examined cases reported in journals, available online, concerning high-risk social web activities. Second, case reports that may generate hypotheses for future clinical studies are in progress (a continually updated cases database, for example). Third, the authors know that no published studies have been conducted but similar data, such as that presented in this work, can be further analyzed in future research. Fourth, this paper serves as an important first step to help to develop a broader area of research, and it underscores several critical situations that have not been presented in official medical databases. Last, very little is known about the psychosocial variables associated with these problematic behaviors, but they represent an emerging mentoring dynamic which is understudied.

### Directions for Future Research

More research is needed in this area. The accidents that occurred during planking activities and the key aspects of influences due to the participation in other activities (eg, drinking games, drinking challenges, planking posted on the Web in different situation and in different areas, neknominations, Web nominations) suggest several critical implications for public and professional education researchers. It is crucial for ED physicians to implement the most effective control measures to reduce the risks associated with fall at heights following challenges to the lowest possible level. Currently, there is no standard definition of planking falls. Falls during planking activities represent an accidental situation related with an incorrect perception of the risks or with an overestimating performance during dangerous activities.

### Conclusions

The injuries and distribution of fractures in the planking falls analyzed here probably originated from the lateral orientation of the body at the moment of impact. Our results highlight the need for further study of the influence of planking in cases of accidental falls from various heights. Previous consumption of alcohol is another problem linked to fatal falls that has not yet been reported for planking. By analyzing injury data, we can identify appropriate types of community prevention approaches, focusing on interventions implementing social changes. Community medical health initiatives may be successful in reducing unintentional injuries; public health begins with the description of a problem and continues with accurate data acquisition, description of injuries and their risk factors, and then builds a surveillance report with the newly acquired data from patients in ED. Several studies have shown that injuries sustained in children after falls are associated with better outcomes, as children have more flexible skeletons, relaxed muscle tone, and a greater proportion of body fat. In clinical and forensic medicine, injuries resulting from falls often become the basis for extensive investigations and autopsy results [13,15]. The general public should receive more information about the new risk of injury and the changing concept of safe behavior by adolescents [6,10]. Education could include information about health programs for adolescents, educators, physicians, and parents. For example, adults should demonstrate positive—and legally compulsory—behavior, always using seat belts while in a car and wearing crash helmets when cycling or biking, but increased knowledge of social network influences is also necessary for the new activity of planking, since this game can involve accidental falls from heights. Decisions on the entire trauma care process should be taken according to a multidisciplinary approach. This paper gives a general overview of the phenomenon of unintentional Web-related trauma and the need for proper education, as human factors contribute 95% to traumatic accidents. Health education and enforcement of legislation are key measures in the implementation of effective strategies.

Key points:

- Internet and social networks are rapidly becoming new ways of communication among adolescents, who change their lifestyles in order to make themselves appear more interesting to their peers, and can potentially influence their behavior. This also involves extreme acts such as planking.
- Planking consists of lying face down on a surface and trying to stay still in balance.
- Planking can sometimes lead to various kinds of trauma, and medical professionals must be aware of these games and practices.

## Abbreviations

CT: computed tomography
ED: emergency department
Eco-FAST: focused abdominal sonography for trauma
ISS: Injury Severity Score
